# The Analysis of the Urban Sprawl Measurement System of the Yangtze River Economic Belt, Based on Deep Learning and Neural Network Algorithm

**DOI:** 10.3390/ijerph17124194

**Published:** 2020-06-12

**Authors:** Huafang Huang, Xiaomao Wu, Xianfu Cheng

**Affiliations:** 1 School of Geography and Tourism, Anhui Normal University, Wuhu 241002, China; huanghf@ahnu.edu.cn; 2 College of Economics & Management, Hefei Normal University, Hefei 230601, China; 3 Anhui Key Laboratory of Natural Disaster Process and Prevention, Wuhu 241002, China; 4 Department of Chemical and Biological Engineering, University of Sheffield, Sheffield, South Yorkshire S10 2TN, UK; wxm19911118@gmail.com; 5 Anhui Province Energy Group Company Limited, Hefei 230011, China

**Keywords:** deep learning, back propagation neural network algorithm, Yangtze River Economic Belt, smart growth evaluation of land use, quantitative growth measurement, urban sprawl measurement system, empirical analysis

## Abstract

In the context of rapid urbanization, the spread of cities in the Yangtze River Economic Belt is intensifying, which has an impact on the green and sustainable development of these cities. It is necessary to establish an accurate urban sprawl measurement system. First, the regulation theory of urban sprawl is explained. According to the actual development situation of cities in the Yangtze River Economic Belt, smart growth theory is selected as the basic regulation method of urban sprawl. Second, the back propagation neural network (BPNN) algorithm under deep supervised learning is applied to construct a smart evaluation model of land use growth. Finally, based on the actual development of cities in the Yangtze River Economic Belt, the quantitative growth measurement method is selected to construct a measurement system of urban sprawl in the Yangtze River Economic Belt, and the empirical analysis is carried out. The training results show that the proposed BPNN smart growth evaluation model, based on deep supervised learning, has good evaluation accuracy, and the error is within the preset range. The analysis of the quantitative growth-based measurement system in the increase of urban construction land shows that the increase in urban construction land area of the Yangtze River Economic Belt from 2014 to 2019 was 78.67 km^2^. Meanwhile, the increases in urban construction land area in different years are different. The empirical results show that the population composition of the Yangtze River Economic Belt and the urban construction area between 2005 and 2019 show a trend of increasing annually; at the same time, urban sprawl development shows a staged characteristic. It is of great significance to apply deep learning fusion neural network algorithm in the construction of the urban sprawl measurement system, which provides a quantitative basis for the in-depth analysis and discussion of urban sprawl.

## 1. Introduction

At present, the urban development in China has entered a rapid stage. Population and land urbanization are important elements in the urbanization process [1,2]. In China, the expansion rate of land urbanization is much faster than that of population urbanization, which leads to the occurrence and development of urban sprawl [3,4]. For the research on the factors affecting urban sprawl, there have been research results worldwide. Chen et al. (2018) completed the construction of the land cover data set by calculating the urban land expansion index in Northeast China from 1990 to 2015; they found that the increase in urban land area is mainly concentrated in coastal areas [5]. Focusing on the phenomenon of serious urban sprawl in collapse-prone areas, Farvacque et al. (2019) proposed an overall quantitative risk analysis procedure based on town scope, which provided a good reference for refined and quantitative land use [6]. Barrington-Leigh and Millard-Ball (2017) explored how to quantify urban sprawl problems and take rash measures to solve urban sprawl problems, revealing the importance of long-term infrastructure decisions [7]. Castillo-Eguskitza et al. (2017) combined land use variables with socio-economic and cultural variables, to explore the evolution of land use and management under autonomous planning in and outside the protected area, which proposed corresponding measures for urban sprawl phenomenon [8]. As shown in the above results, research on urban sprawl mainly focuses on economic, industrial, and policy improvement. After years of development, the measurement method for urban sprawl has already included the single index method, multiple index method, and modern computer information technology. Sarkar and Paul (2017) believed that the measurement and monitoring of urban sprawl urgently need the support of remote sensing software and corresponding technologies; besides, they analyzed and discussed the urban sprawl measurement in the Bardhaman planning area [9]. Bian et al. (2018) studied the factors affecting urban sprawl and the impact of non-tillage environments on animal diversity. The analysis results based on a linear mixed model showed that urban sprawl has a negative impact on arthropods; in addition, the measurement of urban sprawl was analyzed and studied [10]. Zhang et al. (2019) used the super-efficiency data envelopment analysis model to calculate the ecological efficiency of 264 prefecture-level cities in China. Through the empirical test of the panel model and the threshold regression model, as well as the measurement analysis of urban sprawl, the urban sprawl was found to have an influence on both the threshold effects and the ecological efficiency of city size [11]. In summary, currently, research on urban sprawl measurement is abundant, while few research results have been combined with intelligent algorithms and other means. From the perspective of the region where the city is located, the spread of cities in the Yangtze River Economic Belt is very prominent.

On this basis, to expand the analysis and discussion of urban sprawl in the Yangtze River Economic Belt, extend the application of deep intelligent neural network algorithm tools in urban sprawl measurement, thereby achieving reasonable control of urban sprawl in the Yangtze Economic Belt, cities in the Yangtze River Economic Belt are selected as the research objects. To complete the construction of the urban sprawl measurement system in the Yangtze River Economic Belt and provide a reference for the future in-depth study of urban sprawl, the back propagation neural network (BPNN) algorithm under deep supervised learning is introduced to construct the evaluation model of the urban sprawl measurement system. It is hoped to expand the application of deep learning fusion neural network algorithm in the field of urban sprawl.

## 2. Materials and Methods

### 2.1. Theories of Urban Sprawl Regulation

**Figure 1:**
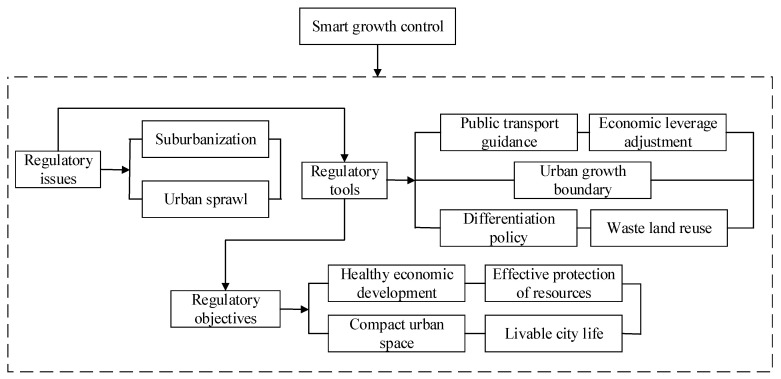
Smart growth regulation mechanism.

To actively respond to a series of negative effects caused by urban sprawl, organizations and scholars have proposed corresponding countermeasures and theories [12,13,14]. Under the current development stage, the corresponding regulation theory proposed for urban sprawl mainly includes the following four aspects. One is the smart growth theory. The urban regulation theory is based on preventing the city from continuing to spread. During the development process of this theory, ten principles are proposed based on regional land use and community construction. It is an effective regulation tool of urban sprawl [15]. The second is the organic concentration theory, which regards the urban development process as an organic combination of concentration and decentralization. However, one of the biggest deficiencies in the application of this theory is that it only describes the spread of the city; accordingly, in contrast, its applications are rare. The third is the compact city theory, which has the characteristic of promoting the sustainable development of society [16,17]. The last theory that has a greater impact on urban sprawl is the new urbanism theory, which proposes corresponding urban sprawl control strategies based on the regional perspective, the town perspective, and the block perspective [18]. Land use is a factor that plays a key role in urban sprawl; in addition, the actual situations of cities in the Yangtze River Economic Belt are also considered. Here, the smart growth theory is chosen as the basic tool for urban sprawl regulation in the Yangtze River Economic Belt, and its regulation mechanism for urban sprawl is shown in Figure 1 below.

### 2.2. BPNN Algorithm Based on Deep Learning

The artificial neural network actually belongs to a form of information processing system. The neural network has the characteristics of self-learning, self-adaptive and parallel processing ability [19,20,21,22]. Thus, it is different from other intelligent algorithm tools. Among the networks, the most widely used is BPNN [23,24]. The composition of the BPNN model includes the input layer, the hidden layer, and the output layer. Specifically, the input layer and the output layer both represent practical problems, and the setting of the hidden layer is mainly based on the actual model and the complexity of the problem [25,26]. BPNN uses deep supervised learning to achieve training and learning [27,28]. If the number of units corresponding to the input layer is n, the input vector is expressed as:

(1) $$x_{i}\left(i=1,2,\ldots ,n\right)$$

Furthermore, if the number of units corresponding to the output layer is q, the output vector corresponding to the input vector is expressed as:

(2) $$y_{t}\left(t=1,2,\ldots ,q\right)$$

Besides, the connection weight between the input layer and the hidden layer is expressed as:

(3) $$w_{ij}\left(i=1,2,\ldots ,n,j=1,2,\ldots ,p\right)$$

The connection weight between the hidden layer and the output layer is expressed as:

(4) $$v_{jt}\left(j=1,2,\;\ldots ,p,t=1,2,\ldots ,q\right)$$

Finally, the threshold corresponding to each unit of the hidden layer is expressed as:

(5) $$\theta_{j}\left(j=1,2,\ldots ,p\right)$$

The threshold corresponding to each unit of the output layer is expressed as:

(6) $$\gamma_{t}\left(t=1,2,\ldots ,q\right)$$

The input and output corresponding to each neuron in the output layer of this neural network algorithm tool can be expressed by the following equations:

(7) $$s_{i}=f\left(\sum_{i=1}^{n}w_{ij}x_{i}+\theta_{j}\right)$$

(8) $$l_{j}=f\left(\sum_{j=1}^{p}v_{jt}s_{i}+\gamma_{t}\right)$$

where: $x_{i}$ represents the input vector, $w_{ij}$ represents the corresponding connection weight between the input layer and the hidden layer, $\theta_{j}$ represents the corresponding threshold value of each unit in the hidden layer, $v_{jt}$ represents the corresponding connection weight between the containing layer and the output layer, $\gamma_{t}$ represents the corresponding threshold of each unit in the output layer, and $\gamma_{t}$ represents that the number of units in the input layer is i. At the same time, in the BPNN algorithm, the expression and calculation corresponding to the Sigmoid function is:

(9) $$f\left(x\right)=\frac{1}{1+e^{-x}}$$

**Figure 2:**
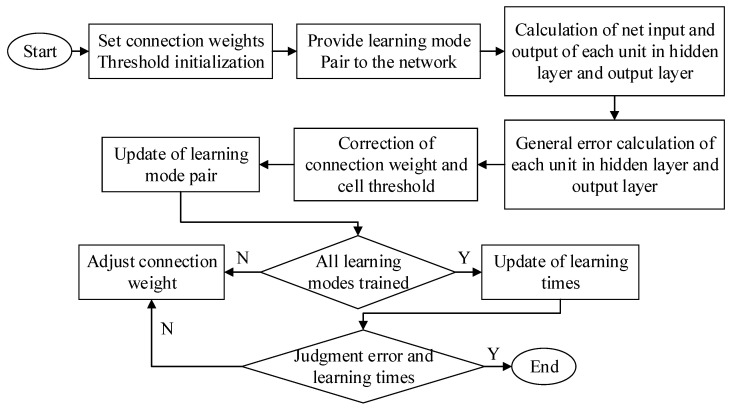
Implementation process of back propagation neural network (BPNN).

The specific implementation process corresponding to the BPNN algorithm is shown in Figure 2 below.

In terms of geographical location, the Yangtze River Economic Belt is a bridge connecting the coast and the inland, which is the backbone of the economic development in China. The Yangtze River Economic Belt includes 11 provinces, such as Jiangsu, Zhejiang, Chongqing, and Yunnan; therefore, this regional cluster has huge development potential. From the perspective of population composition, the population of cities in the Yangtze River Economic Belt accounts for more than 42% of all cities in China. From the perspective of the regional area, the city area of the Yangtze River Economic Belt reaches 21.4% of the country. It is not difficult to find that, in terms of population composition and regional area, cities in the Yangtze River Economic Belt occupy an important position. Before constructing the urban sprawl measurement system of the Yangtze River Economic Belt, the BPNN is utilized as the index model of regional land evaluation. Organically, the deep learning and neural network algorithm are integrated. Then, BPNN is used to design and build the land evaluation model. First, it is necessary to obtain the parameters corresponding to the input layer and output layer of the neural network; second, the land evaluation model is built by determining the hidden layer.

**Figure 3:**
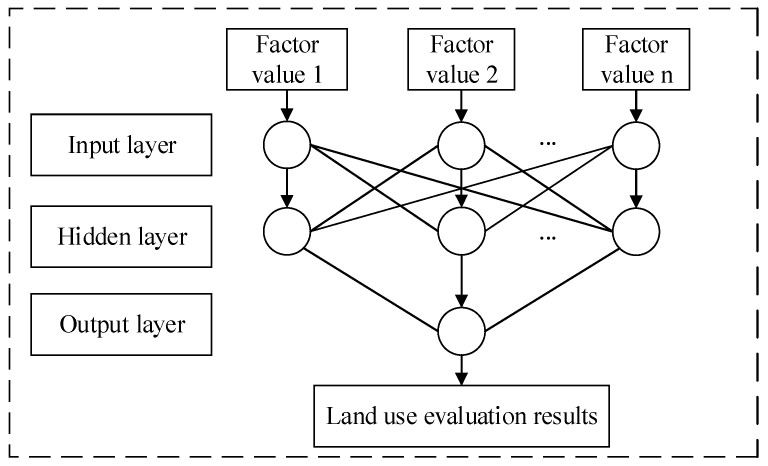
Composition and structure of the deep BPNN land evaluation model.

When using BPNN to construct the land evaluation model, the input layer of the neural network mainly includes various evaluation factors, such as land evaluation factors, standards, and corresponding results. Before the actual evaluation calculation, the index composition of corresponding evaluation factors needs to be standardized to be within the range of [0,1]. The output layer in the neural network mainly includes the evaluation results for the regional land. For the hidden layer in the BPNN, the Sigmoid function is used as an excitation function. On this basis, the composition and structure of the deep BPNN model for the urban land evaluation in the Yangtze River Economic Belt are shown in Figure 3 below.

### 2.3. Smart Growth Model Based on Deep Learning Fusion BPNN Algorithm

**Figure 4:**
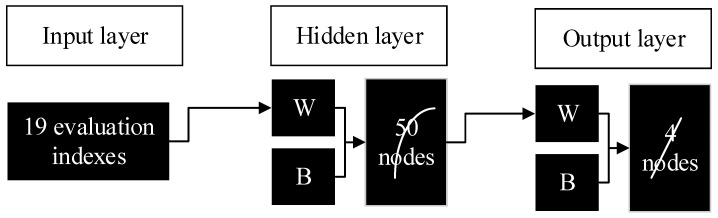
Urban land use smart growth evaluation model.

Here, deep learning and neural network algorithms are organically integrated, and the BPNN algorithm under deep supervised learning is applied to the construction of a smart growth evaluation model for land use in the Yangtze River Economic Belt. Based on the above theories and structural components for BPNN, the constructed deep learning fusion BPNN algorithm builds a smart growth evaluation model of urban land use in the Yangtze River Economic Belt, as shown in Figure 4 below. Corresponding to the BPNN model, the model is mainly composed of the input layer, the hidden layer, and the output layer. The specific evaluation method is: the number of nodes corresponding to the input layer is characterized by the composition of the evaluation index, which corresponds to 19 evaluation indexes, respectively. In the hidden layer, the larger the corresponding number of nodes is, the longer the corresponding operation time will be. On this basis, the number of hidden layer nodes finally determined is 50. For the output layer, the corresponding number of nodes represents the evaluation level of land use in the urban area of the Yangtze River Economic Belt, which is finally determined as 4. As a result, the final composition of the BPNN structure for smart land use growth evaluation is 19 × 50 × 4, and the analysis of the evaluation model is implemented in MATLAB software.

For the training of the evaluation model, the mean square error (MSE) is used as the evaluation index to characterize the training results of the evaluation model [29,30,31]. The specific implementation of the training process is: first, the BPNN is provided with the changes in the population of the Yangtze River Economic Belt and the corresponding areas of the urban construction during 2005–2019, as well as the changes in urban sprawl data. Through continuous training and learning processes, combined with the back propagation of the BPNN, the corresponding weights and thresholds are continuously modified for the network. Therefore, all connection weights between corresponding neurons in the network change, and the error can be gradually reduced until the preset error value is reached. The actual data of the urban population of the Yangtze River Economic Belt and the corresponding area of the urban built-up area from 2005 to 2019 are obtained through network statistical analysis. This takes 15 years, as a research cycle can reveal the evolution and development of the cities in the Yangtze River Economic Belt in recent years.

### 2.4. Construction of Urban Sprawl Measurement System

Accurate measurement of the degree of urban sprawl is an important condition for regulating urban sprawl. However, under the current circumstances, there is no unified measurement selection standard or system [32,33,34]. Considering this situation, based on the above urban sprawl regulation theory, the land use smart growth theory is applied to it. According to the deep supervised learning BPNN model, the model of land use evaluation for the urban area of the Yangtze River Economic Belt is constructed. On this basis, for the construction of the urban sprawl measurement system in the Yangtze River Economic Belt, the measurement standard and measurement index are mainly considered. In terms of the measurement standard constructed for the measurement system of cities in the Yangtze River Economic Belt, the major consideration of the quantitative growth measurement is the phenomenon of increasing the construction area caused by the disorderly expansion of the city [35,36]. Discontinuous measurement, in fact, is a representation of urban sprawl disorder in urban form. Generally, the greater the degree of discontinuity is, the greater the urban sprawl will be [37,38]. Decentralization measurement considers that the faster the urban sprawl develops, the weaker its centrality will be. The measurement of mixing degree assumes that if the other composition conditions do not change, the smaller the mixing degree is, the greater the performance of the corresponding degree of spread will be [39]. Other measurements, such as the accessibility measurement and the open space measurement, also have different measurement standards [40,41].

Urban sprawl is greatly affected by factors such as population growth; in particular, in the cities of the Yangtze River Economic Belt, its influence is greater. Therefore, the quantitative growth measurement is utilized as the standard for evaluating the urban sprawl of the Yangtze River Economic Belt. On this basis, the measurement index corresponding to the urban sprawl measurement system of the Yangtze River Economic Belt is the quantitative growth measurement index, and the expression and calculation of the corresponding sprawl index are shown in the following equation.

(10) $$SI_{i}=50\left\{\left[S_{i}-D_{i}\right]/100+1\right\}$$

where: $SI_{i}$ represents the spread index, $S_{i}$ represents the proportion of the population corresponding to the low-density population in the i regions, and $D_{i}$ represents the proportion of the population corresponding to the high-density population in the i regions. Considered from the perspective of urban construction land, the expression and calculation of the total newly added construction land is shown in the following equation:

(11) $$U_{ab}=\left(U_{b}-U_{a}\right)$$

where: $U_{ab}$ represents the total area of newly added construction land in a certain stage, and $U_{a}$ and $U_{b}$ represent the total area of newly added construction land at the start and end points of the entire research stage. The expression and calculation of the newly added urban construction land expansion speed is shown in the following equation.

(12) $$K=\left(U_{b}-U_{a}\right)/\left(T\cdot U_{a}\right)\cdot 100\%$$

where: T represents the time frame corresponding to the entire research process, and K corresponds to the rate of changes in regional land use during the entire research process. Based on the aforementioned two urban construction land levels, the expression and calculation of the population area elasticity index combining the two are shown in the following equation:

(13) $$I=\left(U_{b}-U_{a}\right)/\left(P_{b}-P_{a}\right)$$

where: I corresponds to the expansion index of construction land, $U_{a}$ and $U_{b}$ correspond to the corresponding area of the land type used at the starting point and the end point of the entire study process, and $P_{a}$ and $P_{b}$ correspond to the population corresponding to the starting and ending points within the entire research process.

To avoid the impact of the non-standard data on the research results, the research data are standardized. Due to the superiority of the range transformation method in data processing, coupled with this method, the effect of the two extreme values on the data is considered. Therefore, this method is selected to complete the standardization of the research data in the measurement system, and the corresponding expression and calculation are as shown in the following equations:

(14) $$y_{ij}=\left(x_{ij}-x_{j}^{0}\right)/\left(x_{j}^{+}-x_{j}^{0}\right)\left(1\leq i\leq m,1\leq j\leq n\right)$$

(15) $$y_{ij}=\left(x_{j}^{+}-x_{ij}\right)/\left(x_{j}^{+}-x_{j}^{0}\right)\left(1\leq i\leq m,1\leq j\leq n\right)$$

where: $x_{j}^{+}$ represents the maximum value corresponding to the positive index, and $x_{j}^{0}$ represents the minimum value.

In summary, to measure the urban sprawl of the Yangtze River Economic Belt, based on the smart growth theory of land use, the quantitative growth measure is used as the measurement standard; combined with the actual situation of the population and land use in the cities of Yangtze River Economic Belt, the population growth and changes in new construction area are taken as specific indicators to measure and evaluate the urban sprawl in the Yangtze River Economic Belt. Different from other regions, for cities in the Yangtze River Economic Belt, the change in population density is a key factor that promotes the sprawl of the cities. Therefore, this influencing factor is crucial and cannot be ignored. Besides, the construction of the urban land area is a direct reflection of the sprawl of regional cities. The change in population growth is the guiding factor for urban sprawl in a region, and the change in the newly built urban area is a characteristic manifestation of urban sprawl. On this basis, the population density change and the urban land area change are chosen as the two major indicators to evaluate the urban sprawl of the Yangtze River Economic Belt.

## 3. Results

### 3.1. Evaluation of the Smart Growth Model

**Figure 5:**
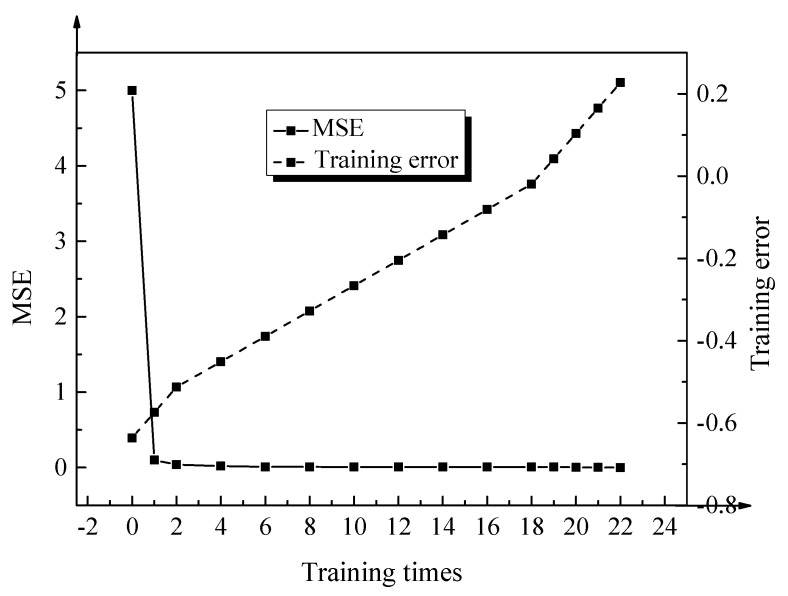
Training results of the land use evaluation model.

Using MSE as the evaluation factor, the training results of the BP neural network land use evaluation model, based on deep supervision, are shown in Figure 5 below.

After analyzing the data changes in the figure, it is found that the constructed evaluation model has achieved the preset accuracy requirements after 22 trainings.

### 3.2. Results of Urban Sprawl Measurement Based on Quantitative Growth Measurement

By using the quantitative growth measurement index, changes in the area of newly built urban construction land in the urban area of the Yangtze River Economic Belt from 2014 to 2019 are selected. 

**Figure 6:**
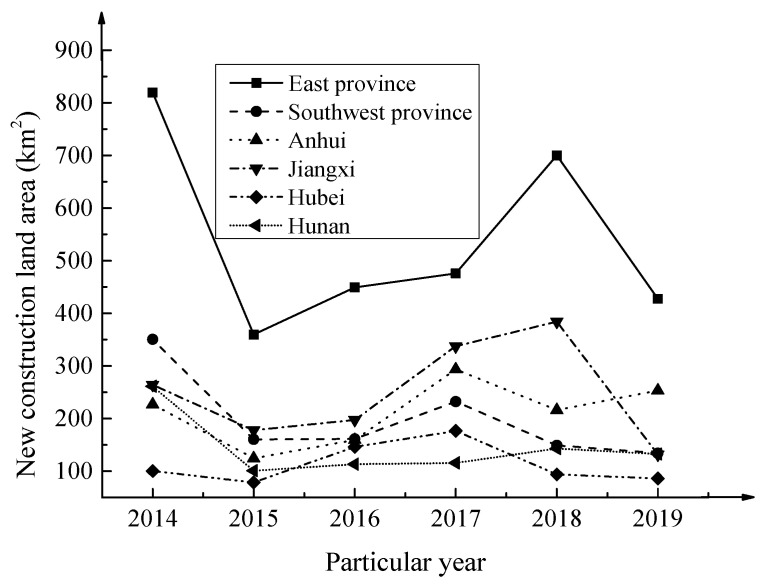
Changes and distribution of new urban construction land area.

The data are utilized as the starting point and the ending point of the entire research process. The changes and distribution of the urban construction land area are shown in Figure 6 below.

After analyzing the data changes in the figure, it is found that the area of urban construction land in the Yangtze River Economic Belt increased by 78.67 km^2^ from 2014 to 2019. At the same time, from a general perspective, the increase in urban construction land in different years is different. The increase in urban construction land area is not very uniform. Among them, the increase was the largest in 2014, and the total urban construction land area in various regions has reached 1761.34 km^2^.

### 3.3. Empirical Analysis of Urban Sprawl Measurement

**Figure 7:**
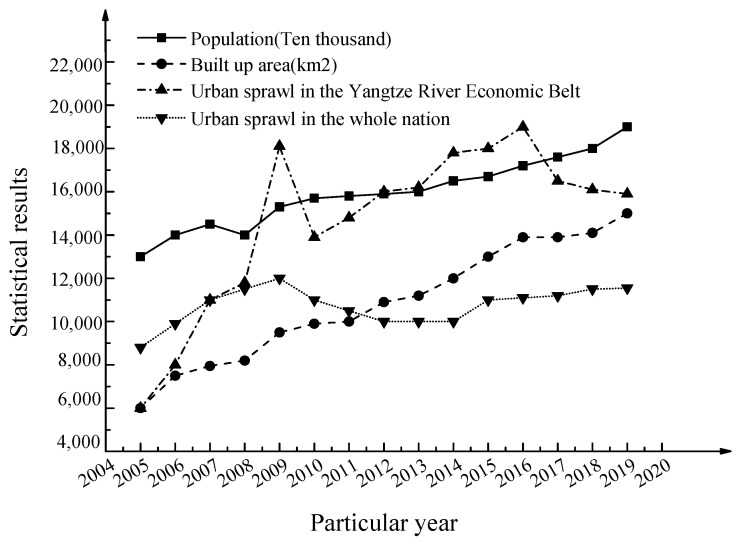
Statistical results of urban sprawl.

Based on the deep supervised learning BPNN evaluation model and the above quantitative growth measurement system, the statistical results of the population composition and area changes of urban construction and urban sprawl in the Yangtze River Economic Belt between 2005 and 2019 are shown in Figure 7 below.

After analyzing the data in the figure, it is found that the population composition of the Yangtze River Economic Belt and the area of the urban construction area show a trend of increasing year by year, which indicates that the urbanization process of cities in the Yangtze River Economic Belt is rapidly occurring. From the perspective of changes in urban sprawl, the urban sprawl of the Yangtze River Economic Belt is above 1 throughout. From 2005 to 2019, the urban sprawl of the Yangtze River Economic Belt is in rapid development. However, at the same time, the urban sprawl of the Yangtze River Economic Belt shows staged development characteristics. The specific manifestations are as follows. From 2005 to 2019, the urban sprawl situation of cities in the Yangtze River Economic Belt shows a process from rapid increase to decrease, and then to a slow increase and slow decrease. From 2005 to 2009, the urban sprawl in the Yangtze River Economic Belt has the fastest growth rate. The sprawl of the cities in the Yangtze River Economic Belt in 2010–2016 shows a slower increase, while in 2009 and 2016–2019, the sprawl of the cities in the Yangtze River Economic Belt shows a decreasing trend. Therefore, for the urban areas of the Yangtze River Economic Belt, the population composition and built-up urban land can indicate the changes in urban sprawl well.

## 4. Discussion

Due to the special geographical location of cities in the Yangtze River Economic Belt, their development and transformation are relatively fast, and the phenomenon of urban sprawl is also more significant. The smart growth theory is selected as the control method for the urban sprawl in the Yangtze River Economic Belt. The BPNN based on deep supervised learning is innovatively applied to the construction of the urban land use the smart growth evaluation model. For the deep supervised learning neural network model, when the corresponding value of MSE is below 0.001 and the corresponding training number is below 10 k, the network model will continue to be passed forward. Besides, when the value corresponding to MSE is not less than 0.001, and the corresponding training times are more than 10 k, it means that the training of the neural network model fails. The above training results show that the land smart growth evaluation model, based on deep supervised learning, has good evaluation accuracy and the error of the model is small, which is of great significance for the construction of the urban sprawl measurement system in the Yangtze River Economic Belt. The smart growth of land use can enable the land resources in the corresponding regions to realize their values effectively. At the same time, it plays an important role in promoting the compact and coordinated development of the corresponding regions and alleviating the unfriendly spread of urban land. The land smart growth evaluation model of deep learning fusion neural network algorithm is applied to the regulation of urban sprawl in the Yangtze River Economic Belt, which is different from the traditional qualitative smart growth evaluation model [42]. The measurement results obtained by both evaluation methods are the same; however, in comparison, the proposed method is more scientific. The evaluation method based on the deep learning fusion neural network algorithm has an active role in effectively regulating the relationship between urban sprawl and economic-social development in the Yangtze River Economic Belt.

At present, there are many measurement methods for urban sprawl, but a unified standard or system has not yet been formed. Considering the actual development of cities in the Yangtze River Economic Belt, the quantity growth measurement method is chosen as the measurement standard for cities in the Yangtze River Economic Belt, which takes the spread index, the total amount of urban construction land, the expansion speed of urban construction land, and the population area elasticity index as the measurement indexes. Based on the changes in the corresponding data of the cities in the Yangtze River Economic Belt in recent years, the sprawl measurement system for the cities in the Yangtze River Economic Belt is constructed. It is found that the urban sprawl in the Yangtze River Economic Belt has significant characteristics, but the changes in urban construction land vary between different years. This may be due to the different emphasis of urban development; thus, there will be differences in the choice of construction land, resulting in the changes in urban construction land of the Yangtze River Economic Belt in different years. The quantitative growth measurement system based on the measurement standard and measurement index pays more attention to changes in urban population density and construction land. This is not applicable to regional cities or cities with low economic development levels. The reason is that for cities under such an equal level of development, population increase is not the main reason for their urban sprawl. Even if there is no population increase, there will still be urban sprawl. Cities in the Yangtze River Economic Belt belong to regions with rapid economic development. Population growth is the key factor that causes their cities to spread or continue to spread. It is reasonable to build an urban sprawl measurement system based on quantitative growth measurements. However, considering this from another perspective, the continuous increase in population will also bring some pressure to the development of the city, leading to the occurrence of disordered urban sprawl. Therefore, it is also very important to regulate the population. Only a reasonable population composition can aid with the development and growth of the cities.

As for the empirical analysis results, compared with the urban spread in recent years in the country, in the first few years of the entire research process, the urban sprawl rate corresponding to the Yangtze River Economic Belt is lower than the national urban sprawl rate. The degree of contagion is not very high, and urban sprawl is mainly concentrated in other regional cities. However, in the latter part of the research process, the spread of cities in the Yangtze River Economic Belt is rising rapidly, and it is much higher than the spread of cities in the country. In recent years, it is in a state of rapid development. The urban sprawl can reflect the development of the corresponding regional economy and the composition of the population, to some extent. Compared with other cities or urban clusters, the cities in the Yangtze River Economic Belt are very prominent in urban sprawl. The spreading phenomenon has some reference values for digging out the development rule of China’s urban sprawl and understanding the corresponding regional characteristics. In recent years, intelligent algorithm tools, including neural network models, have developed rapidly and are widely used. It is of great significance to apply deep learning fusion neural network algorithms to the construction of the urban sprawl measurement system. In recent years, changes in the population composition of the cities in the Yangtze River Economic Belt and changes in the area of newly built cities show that changes in population and building areas have played an important role in the sprawl of cities in the Yangtze River Economic Belt. The sprawling development of cities in the Yangtze River Economic Belt is essentially a microcosm of domestic urban development. Through rational planning and layout, the agglomeration and promotion of new industries are optimized to improve the economic scale and development level of large cities, thereby formulating relevant countermeasures according to the actual situations. At the same time, 6reasonable planning of all aspects of urban production and life is of great significance to delaying urban sprawl and promoting coordinated and orderly economic development.

## 5. Conclusions

By introducing the deep-supervised learning BPNN algorithm into urban sprawl regulation, it is found that the smart algorithm-based land use smart growth evaluation model has good accuracy and applicability in the evaluation of urban sprawl regulation. In recent years, the sprawl of cities in the Yangtze River Economic Belt has shown a gradual change. However, the overall development rate is very fast, which is closely related to the continuous growth of the urban population in the Yangtze River Economic Belt and the increase in the area of new urban land. Affected by various factors, the above analysis is based on the actual situation of the cities in the Yangtze River Economic Belt, while the selected measurement standards and indices have some limitations in the applications of other regional cities with large differences in economic development. Therefore, an in-depth discussion and research on this aspect will be carried out in the future.

## References

[1] Wang Y., Lu N., Luo M., Fan L., Zhao K., Qu J., Guan J., Yuan X. (2019). Enhancement mechanism of fiddlehead-shaped TiO2-BiVO4 type II heterojunction in SPEC towards RhB degradation and detoxification. Appl. Surf. Sci..

[2] Wu T., Perrings C., Kinzig A., Collins J.P., Minteer B.A., Daszak P. (2017). Economic growth, urbanization, globalization, and the risks of emerging infectious diseases in China: A review. Ambio.

[3] Li G., Li F. (2019). Urban sprawl in China: Differences and socioeconomic drivers. Sci. Total Environ..

[4] Feng Y., Wang X. (2019). Effects of urban sprawl on haze pollution in China based on dynamic spatial Durbin model during 2003-2016. J. Clean Prod..

[5] Chen L., Ren C., Zhang B., Wang Z., Liu M. (2018). Quantifying Urban Land Sprawl and its Driving Forces in Northeast China from 1990 to 2015. Sustainability.

[6] Farvacque M., Lopez-Saez J., Corona C., Toe D., Bourrier F., Eckert N. (2019). How is rockfall risk impacted by land-use and land-cover changes? Insights from the French Alps. Glob. Planet. Chang..

[7] Barrington-Leigh C., Millard-Ball A. (2017). More connected urban roads reduce US GHG emissions. Environ. Res. Lett..

[8] Castillo-Eguskitza N., Rescia A.J., Onaindia M. (2017). Urdaibai Biosphere Reserve (Biscay, Spain): Conservation against development?. Sci. Total Environ..

[9] Sarkar A., Paul B. (2017). Corrigendum to “The global menace of arsenic and its conventional remediation—A critical review” [Chemosphere 158 (September) (2016) 37–49]. Chemosphere.

[10] Bian Z.X., Wang S., Wang Q.B., Yu M., Qian F.K. (2018). Effects of urban sprawl on arthropod communities in peri-urban farmed landscape in Shenbei New District, Shenyang, Liaoning Province, China. Sci. Rep..

[11] Zhang Q., Zhang H., Zhao D., Cheng B., Yu C., Yang Y. (2019). Does Urban Sprawl Inhibit Urban Eco-Efficiency? Empirical Studies of Super-Efficiency and Threshold Regression Models. Sustainability.

[12] Feng B., Sun K., Chen M., Gao T. (2020). The Impact of Core Technological Capabilities of High-Tech Industry on Sustainable Competitive Advantage. Sustainability.

[13] Yan X., Chen M., Chen M.-Y. (2019). Coupling and Coordination Development of Australian Energy, Economy, and Ecological Environment Systems from 2007 to 2016. Sustainability.

[14] Lee C. (2019). Impacts of urban form on air quality: Emissions on the road and concentrations in the US metropolitan areas. J. Environ. Manag..

[15] Hong W. (2017). Solving the 5G Mobile Antenna Puzzle: Assessing Future Directions for the 5G Mobile Antenna Paradigm Shift. IEEE Microw Mag..

[16] Yokoyama M., Matsuo K., Tanaka T., Sadohara S. (2018). Designing and evaluating land use scenario with effective sea breeze use: Study on land use scenarios of compact city with mitigating urban warming effect in Kanagawa prefecture Part2. J. Environ. Eng..

[17] Merhav N., Sason I. (2019). An Integral Representation of the Logarithmic Function with Applications in Information Theory. Entropy.

[18] Brown A. (2019). The Black Skyscraper: Architecture and the Perception of Race. Technol. Cult..

[19] Kachba Y., Chiroli D.M., Belotti J.T., Alves T.A., de Souza Tadano Y., Siqueira H. (2020). Artificial Neural Networks to Estimate the Influence of Vehicular Emission Variables on Morbidity and Mortality in the Largest Metropolis in South America. Sustainability.

[20] Pei J., Liu W. (2019). Evaluation of Chinese Enterprise Safety Production Resilience Based on a Combined Gray Relevancy and BP Neural Network Model. Sustainability.

[21] Ngoc Q.T., Lee S., Song B.C. (2020). Facial Landmark-Based Emotion Recognition via Directed Graph Neural Network. Electronics.

[22] Polat H., Danaei Mehr H. (2019). Classification of Pulmonary CT Images by Using Hybrid 3D-Deep Convolutional Neural Network Architecture. Appl. Sci..

[23] Kim S.-K., Huh J.-H. (2020). Artificial Neural Network Blockchain Techniques for Healthcare System: Focusing on the Personal Health Records. Electronics.

[24] Fan H., Han M., Li J. (2019). Image Shadow Removal Using End-To-End Deep Convolutional Neural Networks. Appl. Sci..

[25] Liu X., Jing W., Zhou M., Li Y. (2019). Multi-Scale Feature Fusion for Coal-Rock Recognition Based on Completed Local Binary Pattern and Convolution Neural Network. Entropy.

[26] Wang B., Gu X., Ma L., Yan S. (2017). Temperature error correction based on BP neural network in meteorological wireless sensor network. Int. J. Sens. Netw..

[27] Wang J., Fang K., Pang W., Sun J. (2017). Wind Power Interval Prediction Based on Improved PSO and BP Neural Network. J. Electr. Eng. Technol..

[28] Davarzani S., Saucier D., Peranich P., Carroll W., Turner A., Parker E., Middleton C., Nguyen P., Robertson P., Smith B. (2020). Closing the Wearable Gap—Part VI: Human Gait Recognition Using Deep Learning Methodologies. Electronics.

[29] Inatsu Y., Imori S. (2018). Model selection criterion based on the prediction mean squared error in generalized estimating equations. Hiroshima Math. J..

[30] Xia Y., Mandic D.P. (2018). Augmented Performance Bounds on Strictly Linear and Widely Linear Estimators with Complex Data. IEEE Trans. Signal Process..

[31] Mathews A.B., Devadhas G.G. (2019). Non linearity mitigation and dispersion reduction using Bussgang theorem, modified MSE and improved MLE equalizers. Microprocess. Microsyst..

[32] Altieri L., Cocchi D., Roli G. (2019). Measuring heterogeneity in urban expansion via spatial entropy. Environmetrics.

[33] Oreopoulos J., Gray-Owen S.D., Yip C.M. (2017). High Density or Urban Sprawl: What Works Best in Biology?. ACS Nano.

[34] Zheng Y., Wang L., Chen H., Lv A. (2019). Does the Geographic Distribution of Manufacturing Plants Exacerbate Groundwater Withdrawal? -A case study of Hebei Province in China. J. Clean Prod..

[35] Iwasaki M., Qi G., Endo Y., Pan Z., Yamashiro T. (2019). Quantity changes in Pseudomonas species in dairy manure during anaerobic digestion at mesophilic and thermophilic temperatures. J. Mater. Cycles Waste.

[36] Kumar R.R., Stauvermann P.J., Shahzad S.J.H. (2017). Can technology provide a glimmer of hope for economic growth in the midst of chaos? A case of Zimbabwe. Qual. Quant..

[37] Maxim S. (2018). On “discontinuous” continuity equation and impulsive ensemble control. Syst. Control Lett..

[38] Fiengo Pérez F., Sweeck L., Elskens M., Bauwens W. (2017). A discontinuous finite element suspended sediment transport model for water quality assessments in river networks. Hydrol. Process..

[39] Kang P.K., Bresciani E., An S., Lee S. (2019). Potential impact of pore-scale incomplete mixing on biodegradation in aquifers: From batch experiment to field-scale modeling. Adv. Water Resour..

[40] Ding J., Zhang Y., Li L. (2018). Accessibility Measure of Bus Transit Networks. IET Intell. Transp. Syst..

[41] Golmohammadi R., Aliabadi M., Nezami T. (2017). An Experimental Study of Acoustic Comfort in Open Space Banks Based on Speech Intelligibility and Noise Annoyance Measures. Arch. Acoust..

[42] Noland R.B., Weiner M.D., DiPetrillo S., Kay A.I. (2017). Attitudes towards transit-oriented development: Resident experiences and professional perspectives. J. Transp. Geogr..

